# Effect of Graphene Oxide/Graphene Hybrid on Mechanical Properties of Cement Mortar and Mechanism Investigation

**DOI:** 10.3390/nano10010113

**Published:** 2020-01-07

**Authors:** Hongfang Sun, Li Ling, Zhili Ren, Shazim Ali Memon, Feng Xing

**Affiliations:** 1 Guangdong Provincial Key Laboratory of Durability for Marine Civil Engineering, College of Civil and Transportation Engineering, Shenzhen University, Shenzhen 518060, China; sunhf03@szu.edu.cn (H.S.); popwii90@gmail.com (L.L.); renzhili219@gmail.com (Z.R.); 2 Department of Civil Engineering and Environmental Engineering, School of Engineering and Digital Sciences, Nazarbayev University, Astana 010000, Kazakhstan; shazimalimemon@gmail.com

**Keywords:** graphene oxide, graphene, dispersibility, cement mortar, mechanical properties

## Abstract

This paper evaluated the effect of graphene oxide/graphene (GO/GR) hybrid on mechanical properties of cement mortar. The underlying mechanism was also investigated. In the GO/GR hybrid, GO was expected to act as a dispersant for GR while GR was used as reinforcement in mortar due to its excellent mechanical properties. For the mortar specimen, flexural and compressive strength were measured at varied GO to GR ratios of 1:0, 3:1, 1:1, 1:3, and 0:1 by keeping the total amount of GO and GR constant. The underlying mechanism was investigated through the dispersibility of GR, heat releasing characteristics during hydration, and porosity of mortar. The results showed that GO/GR hybrid significantly enhanced the flexural and compressive strength of cement mortars. The flexural strength reached maximum at GO:GR = 1:1, where the enhancement level was up to 23.04% (28 days) when compared to mortar prepared with only GO, and up to 15.63% (7 days) when compared to mortar prepared with only GR. In terms of compressive strength, the enhancement level for GO:GR = 3:1 was up to 21.10% (3 days) when compared with that of mortar incorporating GO only. The enhancement in compressive strength with mortar at GO:GR = 1:1 was up to 14.69% (7-day) when compared with mortar incorporating GR only. In addition to dispersibility, the compressive strength was also influenced by other factors, such as the degree of hydration, porosity, and pore size distribution of mortar, which made the mortars perform best at different ages.

## 1. Introduction

Cementitious materials are the most widely used construction materials worldwide [1]. However, cementitious materials are generally brittle and susceptible to cracking [2]. In the last decade, nano-materials have been introduced to cementitious materials to delay the nucleation and growth of cracks at nanoscale and to prevent the propagation of cracks to micro-and macro-levels [3].

Graphene (GR) as a new member of nano-material family, ideally, is able to significantly reinforce cement-based materials due to its excellent mechanical properties. The tensile strength and elastic modulus of GR is estimated to be 100 GPa and 1 TPa, respectively [4,5]. However, due to its poor dispersion capability of GR arising from the surface hydrophobicity and strong interlayer van der Waals forces [6], their use in cementitious materials has remained limited. Additionally, its performance in cement based composite needs to be improved. Wang [7] reported that the flexural strength of GR-cement composite showed a more remarkable increase when compared to the compressive strength. The incorporation of 0.05 wt% GR in cement paste enhanced the 28-day flexural strength and compressive strength by 16.8% and 1.3%, respectively. Hou et al. [8] added 0.16% of GR (by weight of cement) into cement paste and found that the 28-day compressive and flexural strength decreased by 3.36% and 10.59%, respectively. Cao et al. [9] observed that by adding GR at 0.02 wt% by weight of cement, the enhancement in flexural and compressive strength at 28 days was up to 32.0% and 20.3%, respectively. Thereafter, effective dispersion of GR is necessary to enhance the performance of cement-based materials.

Graphene oxide (GO), although having a similar structure as that of GR, has oxygen-containing functional groups linked to carbon atoms [10,11,12]. The functional groups make GO hydrophilic and highly dispersible in aqueous solution [13,14]. Additionally, the high surface area could be beneficial for improving bonding between graphene sheets and hydration products of cement when incorporated to cement-based materials [15]. Therefore, the enhancement in mechanical properties of GO modified cementitious materials was observed. For example, Lu et al. [16] reported that the addition GO at 0.05 wt% of cement can improve the compressive and flexural strength of the magnesium potassium phosphate cement (MKPC) paste by 6.8% and 8.3%, respectively. Lv et al. [17] added GO sheets in cement paste at dosages of 0.03% (by weight of cement) and found that the flexural and compressive strengths increased by 52.4% and 34.3%, respectively. Zhao et al. [18] found that at 28 days the compressive and flexural strengths of cement paste containing 0.022% GO were greater than those of plain cement paste by 17.68% and 22.55%, respectively. However, it is also reported that the further increase in mechanical properties is difficult which may be due to decreased tensile strength of GO when compared with GR owing to the breakage of carbon chains by functional groups [19,20]. Therefore, the function of GO in cementitious materials needs to be explored further.

In recent years, researchers have found that GO can be used as a dispersant due to its high surface area, which in turn, improves the dispersibility of a variety of nano-materials. Xu et al. [21] reported that multi-walled carbon nanotubes (MWNT) were efficiently dispersed in water when using GO as dispersant. Cheng et al. [22] found that carbon nanotubes (CNTs) were highly dispersed by GO when compared with surfactants. The excellent reinforcing capabilities of the hybrid GO/CNTs were also demonstrated by the enhanced fracture resistance properties of the cementitious matrix. Hong et al. [23] found that the incorporation of GO greatly enhances the aqueous dispersion of a robust Cu_2_O nano-catalyst. From these studies, it can be speculated that GO might act as dispersant to improve the dispersibility of GR in water, so that the excellent mechanical properties of GR can be utilized to further enhance the crack resistance of cement-based materials. According to authors’ best knowledge, this idea has not been previously reported in literature.

Therefore in this research, GO/GR hybrid was prepared at varied ratios to evaluate its influence on the mechanical properties of cement mortars. The underlying mechanism was also investigated. Thus, the work in this research explored the new role of nano-materials in the application of cementitious materials.

## 2. Materials and Methods

### 2.1. Preparation of Materials

In order to investigate the effect of GO/GR hybrid on cement based mortar and understand the mechanism involved, the material’s preparation process would be described from three aspects.

1. Synthesis of GO in GO/GR hybrid.
2. Preparation of GO/GR hybrid.
3. Preparation of cement mortars incorporating GO/GR hybrid.

#### 2.1.1. Synthesis of GO

**Figure 1:**
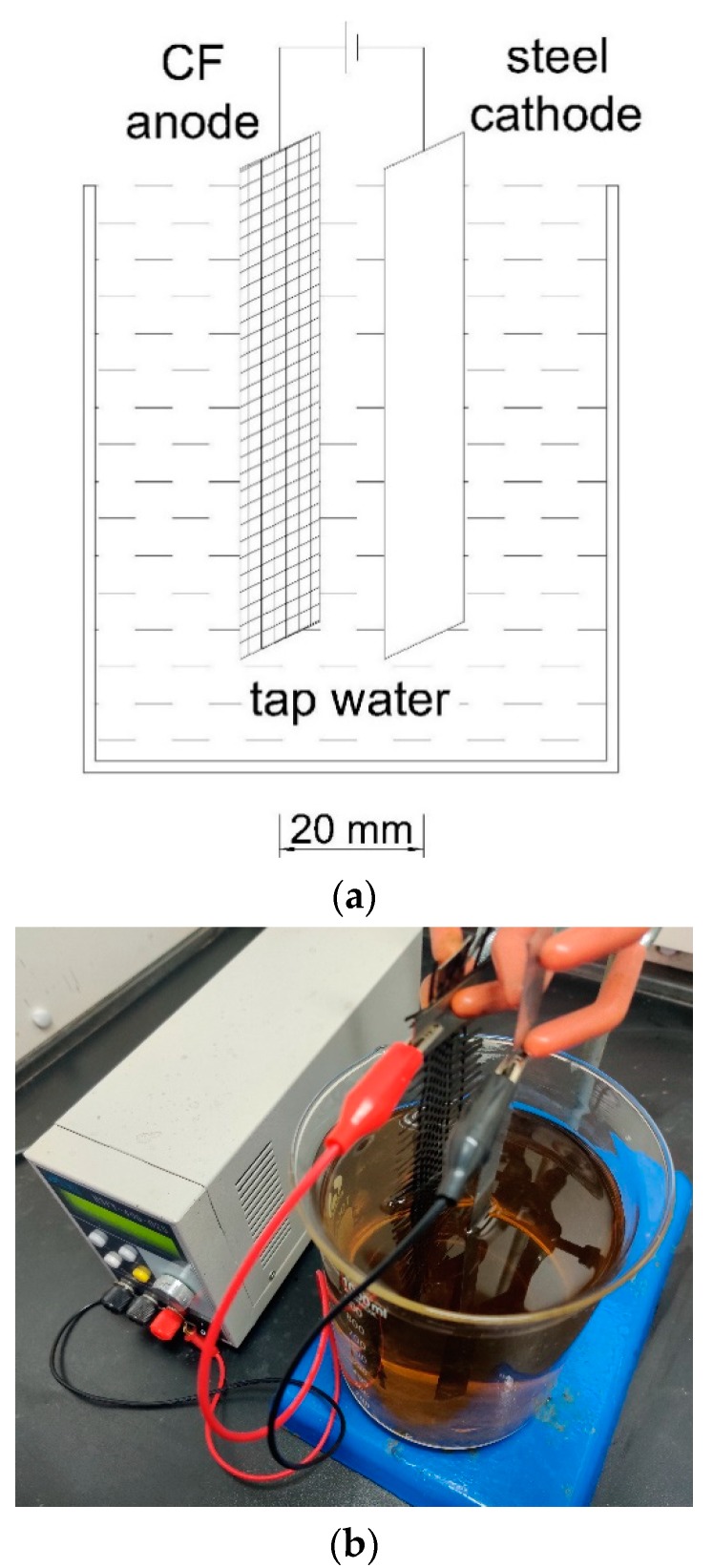
Schematic view (**a**) and photograph (**b**) of the electrochemical system to prepare oxide/grapheme (GO).

In this research, GO in GO/GR hybrid was synthesized through an electrochemical method [24,25]. The synthesis system consisted of an anode in the form of carbon fiber sheet, a cathode in the form of stainless steel sheet, electrolytes (tap water), and a DC power supply (Figure 1). The carbon fiber sheet having size of 200 mm (length) × 40 mm (width) × 0.22 mm (thickness) and weight per area of 208 g/m^2^ was purchased from CA.BEN composite Co., Ltd. (Guangzhou, China) while the size of stainless steel sheet for cathode was 200 mm (length) × 15 mm (width) × 0.5 mm (thickness). Both anode and cathode were immersed in electrolyte up to a depth of 130 mm, and the distance between them was 20 mm. During the synthesizing process, the current was kept constant at 10 mA (current density of 0.97 A/m^2^ of the anode). After 30 days, the electrolyte became black to form the final well-dispersed GO solution.

**Figure 2:**
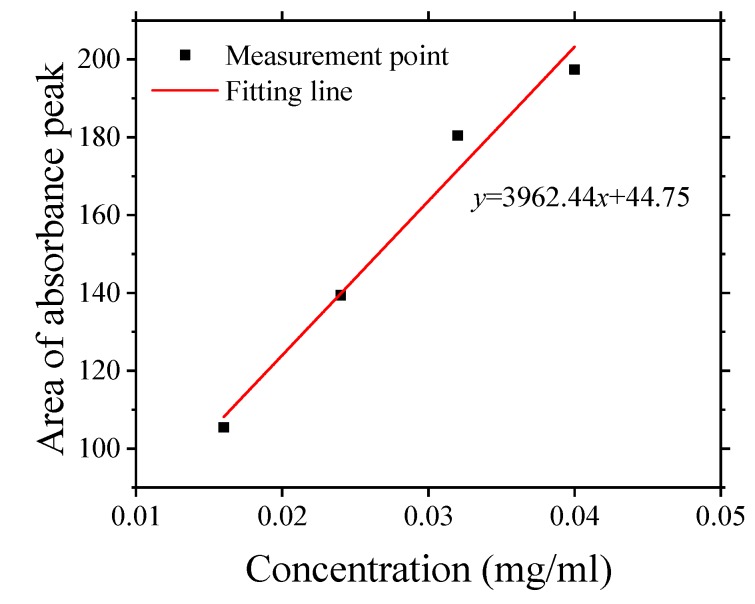
The relationship between the concentration of graphene oxide (GO) solution purchased commercially and the area of absorption peak.

In order to determine the concentration of GO in the prepared solution, ultraviolet (UV) spectrometer was used. For this purpose, GO with a known concentration was purchased commercially (CGO) and four CGO solutions with gradient concentrations of 0.016, 0.024, 0.032, 0.040 mg/mL, respectively, were prepared. and four CGO solutions with gradient concentrations of 0.016, 0.024, 0.032, 0.040 mg/mL, respectively, were prepared. Then, the area of absorption peak in spectra within the wavelength of 190–500 nm was observed. Thereafter, linear curve fitting was done to establish the relationship between the concentration of CGO and the area of absorbance peak as shown in Figure 2. The data obtained was compared with the UV spectrum of prepared GO (diluted) and finally the concentration of GO was determined to be 2.47 mg/mL.

GO powder was also prepared for morphology and composition analyses purpose by evaporating water from GO solution at 70 °C.

#### 2.1.2. Preparation of GO/GR Hybrid

For the preparation of GO/GR hybrid, GR powder was purchased from Tanfeng Graphene Technology Ltd. (Suzhou, China). with purity exceeding 95%, thickness of 3–5 nm, slice diameter <20 µm, layer number of 1–5. In this phase, GO/GR hybrid was prepared at different ratios varying from 1:0 to 0:1 by keeping the total amount constant (Table 1). The deionized water (GO:GR = 0:0) was also added as the control specimen. Each hybrid was dispersed by using ultrasonic device for 10 min at 400 W.

#### 2.1.3. Preparation of Cement Mortar with GO/GR Hybrid

Cement mortars were prepared by mixing cement, sand, superplasticizer (SP), and GO/GR hybrid to evaluate the effect of GO/GR hybrid on mortar properties. The cement (P.I 42.5R) having properties as shown in Table 2, was produced by China United Cement Ltd. (Juye, China). The sand having particle size ranging from 0.08 to 2 mm was produced by Xiamen ISO sand Ltd. (Xiamen, China). In addition, polycarboxylic superplasticizer with solid content of 55% was added to improve the dispersibility of GO/GR hybrid in cement mortar. The water to cement ratio (w/c) and cement to sand ratio (c:s) were 0.35 and 1:3, respectively, while the dosage of SP was kept at 0.6% (relative to the mass of cement). Each prepared cement mortar was molded into a rectangle having size of 40 mm × 40 mm × 160 mm. After 24 h, the molds were removed and then the samples were cured at temperature of 20 ± 3 °C and relative humidity of 95% until the desired age to testing. For each mix, three samples were prepared and tested.

### 2.2. Testing

The morphology and characterization of self-synthesized GO was performed by transmission electron microscopy (TEM), Fourier transform infrared spectroscopy (FTIR), Raman, and UV spectrometry techniques. The morphology of GO was observed by TEM technique using JEM-2100F Field Emission Electron Microscope (JEOL, Tokyo, Japan) at accelerating voltage of 200 kV. The sample was prepared by loading a drop of GO solution onto a carbon coated Cu TEM mesh grid and fully evaporating the water at room temperature for TEM observation. The composition of GO was tested by Raman technology using Renishaw InVia Reflex Raman Microscope (Renishaw, Hoffman Estates, IL, USA) equipped with a 50× objective, and excitation wavelengths of 514 nm (Argon Ion laser). The samples were prepared by spreading GO powder on double-sided tape which was mounted on a glass slide. The functional groups of GO and GR were determined by using Perkin-Elmer Spectrum 100 FTIR spectrometer (Perkin-Elmer, Norwalk, CT, USA) at 8-cm^−1^ resolution in the range 4000–700 cm^−1^. The FTIR spectra were obtained in ATR mode and for each sample, three readings were taken. The concentration of GO solution was obtained by using Perkin Elmer Lambda 750 UV-VIS spectrophotometer (Perkin Elmer, Waltham, MA, USA) in 190–500 nm wavelength range. Here, tap water was used for baseline correction.

The mechanical properties (flexural and compressive strength) of cement mortar incorporating GO/GR hybrid were determined by using YZH-300.10 Computerized electronic universal testing machine (LUDA, Shaoxing, China). For each mix, three specimens were tested at 3, 7, and 28 days. In the flexural strength test, 40 mm × 40 mm × 160 mm blocks were tested at a pressure rate of 0.05 kN/s while in the compressive strength test, 40 mm × 40 mm × 40 mm blocks were utilized at a pressure rate of 2.4 kN/s.

The heat flow and cumulative heat released by the cement during the hydration was monitored by Calorimetric technique using TAM Air eight-channel Thermal Microcalorimeter (TA instrument, New Castle, DE, USA). The cements in each ampoule (capacity of 20 mL) were mixed with GO/GR hybrid by using a syringe to have a w/c of 0.35 and vibrated for 30 s in order to have homogeneous mixing. Then, the vials/container were immediately placed in the calorimeter chamber. The experiments were performed at 23 °C. As a result of the hydration process, heat is generated and the rate of heat production is continuously monitored as a function of time (for 168 h).

The pore size distribution of cement mortar was measured by mercury intrusion porosimetry (MIP) conducted on AutoPore IV 9500 (Micromeritics, Norcross, GA, USA). For full-scale intrusion volume, the intrusion accuracy was ±1% and the pressure range was 0.10–30,000.00 psia. The output obtained was porosity, pore diameter distribution, and proportion of pore diameter under 50 nm.

## 3. Results

### 3.1. Properties of GO

**Figure 3:**
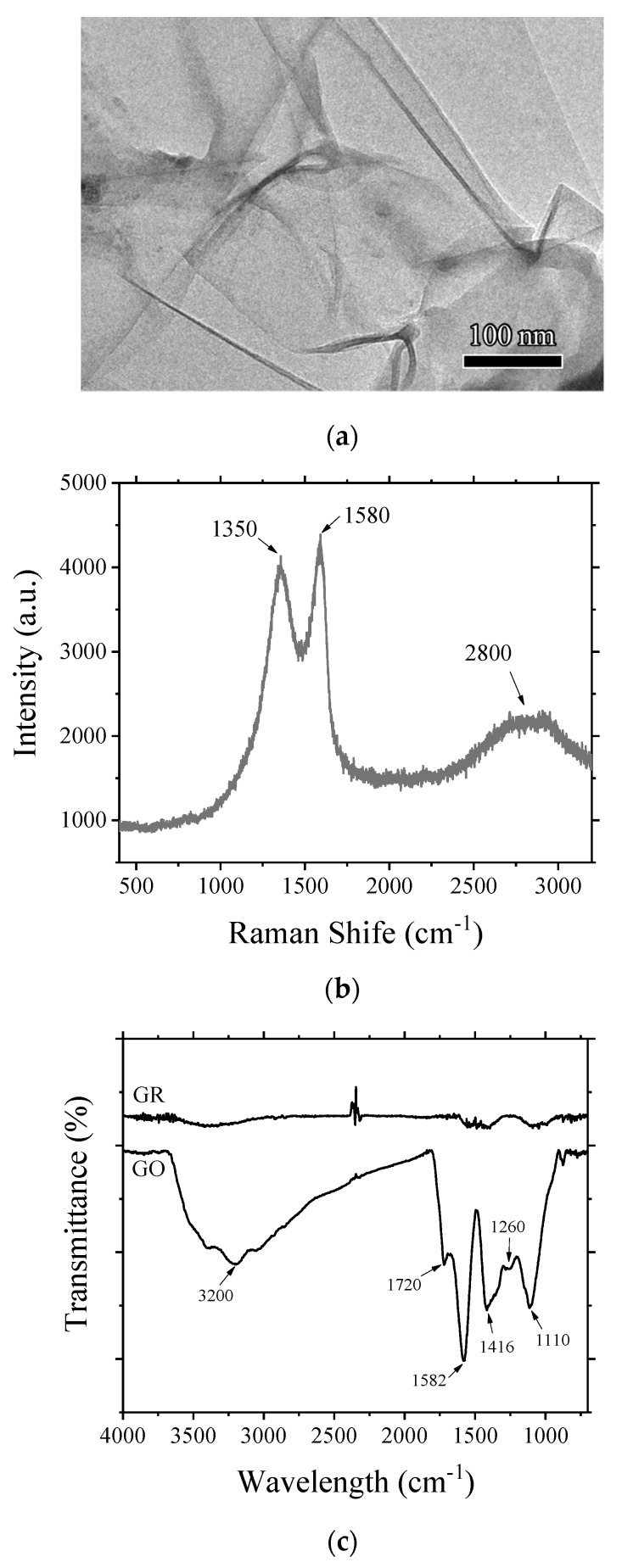
TEM image (**a**) and Raman spectrum (**b**) of GO; (**c**) FTIR spectra of GR and GO.

In this section, the results of characterization of GO are presented. The morphology of GO observed by using TEM is presented in Figure 3a. It can be seen that the prepared GO has a typical 2D thin-film microstructure, which is similar to the morphology of GO produced by traditional Hummer’s method [26,27].

Raman technique was used to determine the composition of GO. The results presented in Figure 3b show peaks at 1350 cm^−1^, 1580 cm^−1^, and 2800 cm^−1^, which are typical characteristic peaks of GO. The D peak at 1350 cm^−1^ is used to characterize defects caused by functional groups in graphite microcrystals. The presence of D peak in the spectrum indicates that the prepared powder was graphite oxide. As far as G peak is concerned, it was observed at 1580 cm^−1^ and represents the presence of perfect crystalline part. The 2D peak at 2800 cm^−1^ has a large peak width, indicating that the prepared GO has multiple layers [28].

FTIR was used to determine the functional groups. The results of FTIR spectra presented in Figure 3c indicate that in comparison to GR, GO carried more functional groups, including carboxyl groups (–COOH, 3200, 1582 cm^−1^), carbonyl groups (C=O, 1720 cm^−1^), hydroxyl groups (–OH, 1416 cm^−1^), and ether bonds (–C–O–C–, 1260, 1110 cm^−1^) [29,30,31]. The existence of large amounts of functional groups in GO resulted in excellent hydrophilicity, improved stability in aqueous solutions, and showed potential as dispersant for other nano-materials.

### 3.2. Mechanical Properties of Cement Mortar with GO/GR Hybrid

**Figure 4:**
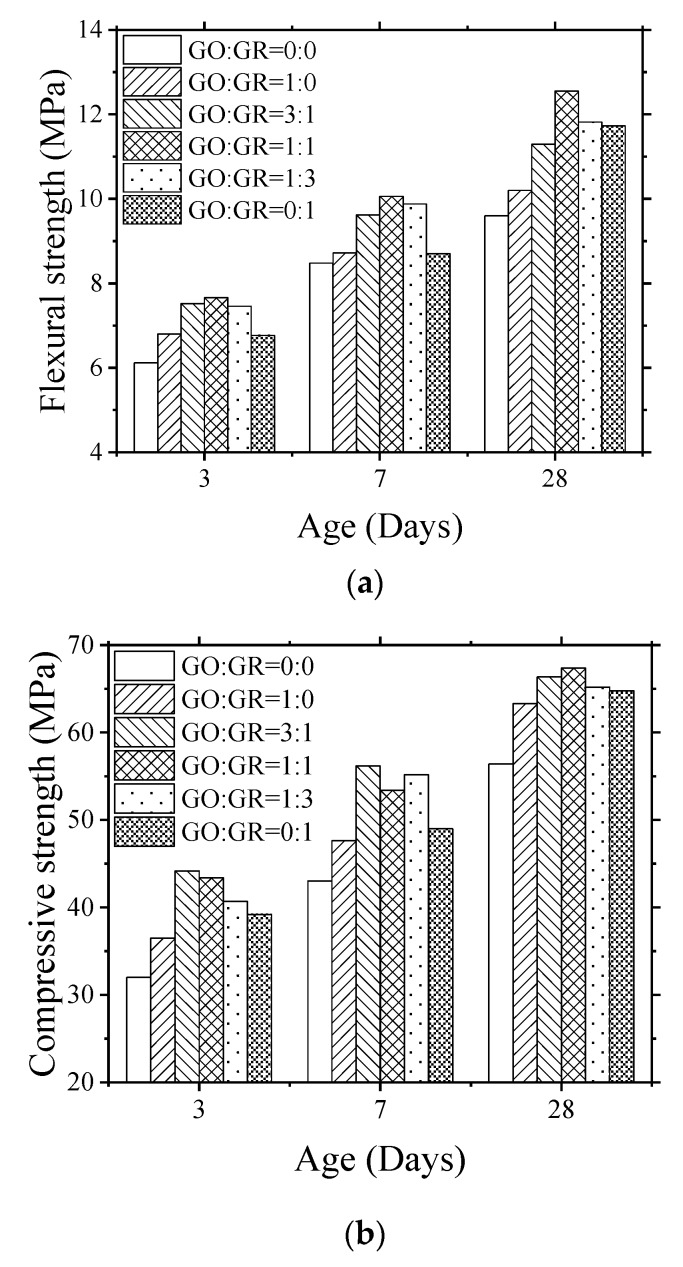
Mechanical properties of cement mortars incorporating GO/GR hybrid with varied ratios. (**a**) Flexural strength; (**b**) Compressive strength.

In order to investigate the influence of GO/GR hybrid on the mechanical properties of cement mortar, flexural and compressive strength of mortar at the age of 3, 7, and 28 days of mortar was tested. The results are presented in Figure 4, Table 3 and Table 4. It can be seen that compared to the control specimen (GO:GR = 0:0), specimens containing only GO (GO:GR = 1:0) or GR (GO:GR = 0:1) showed higher flexural and compressive strength at all the ages of testing. In comparison to control specimen, the specimen prepared with only GO and GR showed an increase of flexural strength by 6.25% and 22.19%, respectively, at the age of 28 days, meanwhile the percentage increase of compressive strength was found to be 12.23% and 14.89%, respectively. This indicates that GR has much better mechanical properties than GO and could effectively reinforce the mortar.

It is interesting to note that when GO/GR hybrid was added to cement mortar, both the flexural and compressive strengths were further increased. The flexural strength enhancement reached maximum at GO:GR = 1:1, where the 3-day, 7-day, and 28-day strength increased by 25.16%, 18.63%, and 30.73%, respectively, when compared with control specimen. As far as compressive strength is concerned, it reached maximum value at GO:GR = 3:1 at the age of 3 and 7 days. However, at 28 days, strength enhancement was maximum at GO:GR = 1:1.

Following are the potential reasons of flexural strength enhancement of GO/GR hybrid cement based mortar. (1) Dispersion of GR in GO solution (evaluated by the observation made after 24 h rest period as demonstrated in next section); and (2) the dosage of GR. Some of the possible reasons of compressive strength enhancement are [32,33,34] (1) dispersion of GO/GR hybrid; (2) degree of hydration of cement (evaluated by calorimetry); and (3) filler effect of nano-materials (evaluated by pore size distribution). Therefore, in the following sections, the mechanism of strength enhancement would be investigated based on abovementioned aspects.

### 3.3. Dispersion of GR in GO Solution

**Figure 5:**
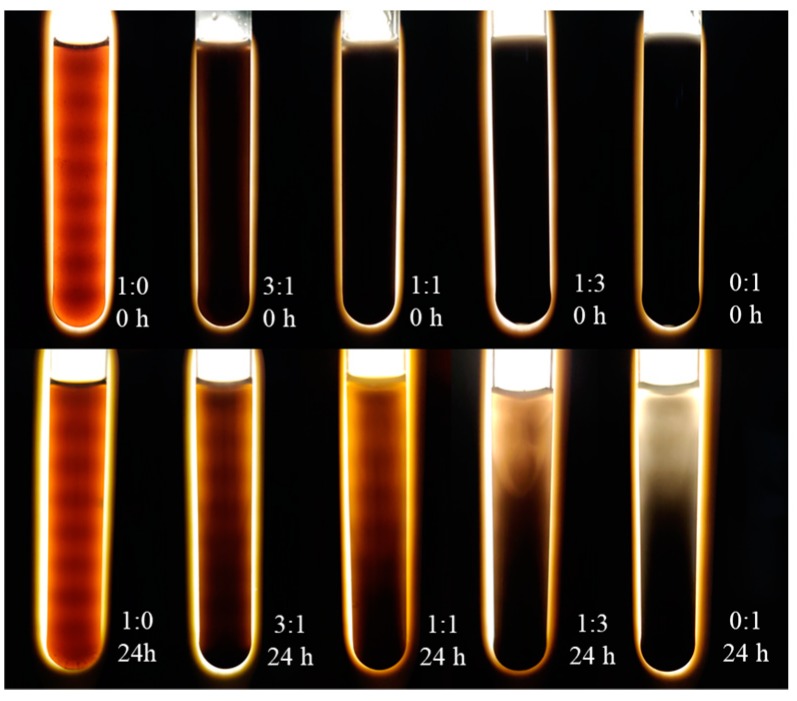
Dispersion of GO/GR hybrid just stirred and stood for 24 h.

In order to determine the dispersibility of GR in GO solvent, GO and GR as well as SP were mixed according to the ratios mentioned in Table 1. The samples were observed immediately after ultrasonication and after 24 h rest period. The dispersion was observed with the assistance of LED light. The results are presented in Figure 5.

It can be seen that the immediately after stirring (0 h), all the prepared specimens showed good dispersion since the color along the test tubes is uniform. After the rest period of 24 h, the difference in the color of specimens appeared. The color of the specimen with only GO (GO:GR = 1:0) is still uniform without any bleaching, indicating a good dispersion of GO in water. On the contrary, the specimen with only GR (GO:GR = 0:1) showed a fade color at the upper part of tube and significant segregation occurred indicating the dispersion of GR is not good in water. In this specimen, almost all the GR powder settled to the bottom of the tube. When GO and GR was mixed at various ratios (3:1, 1:1, and 1:3), the color difference of solution in the upper and lower parts of test tube became less significant with the increase of GO dosage. This indicated that GO solution could effectively improve the dispersion of GR in water.

### 3.4. Calorimetry

**Figure 6:**
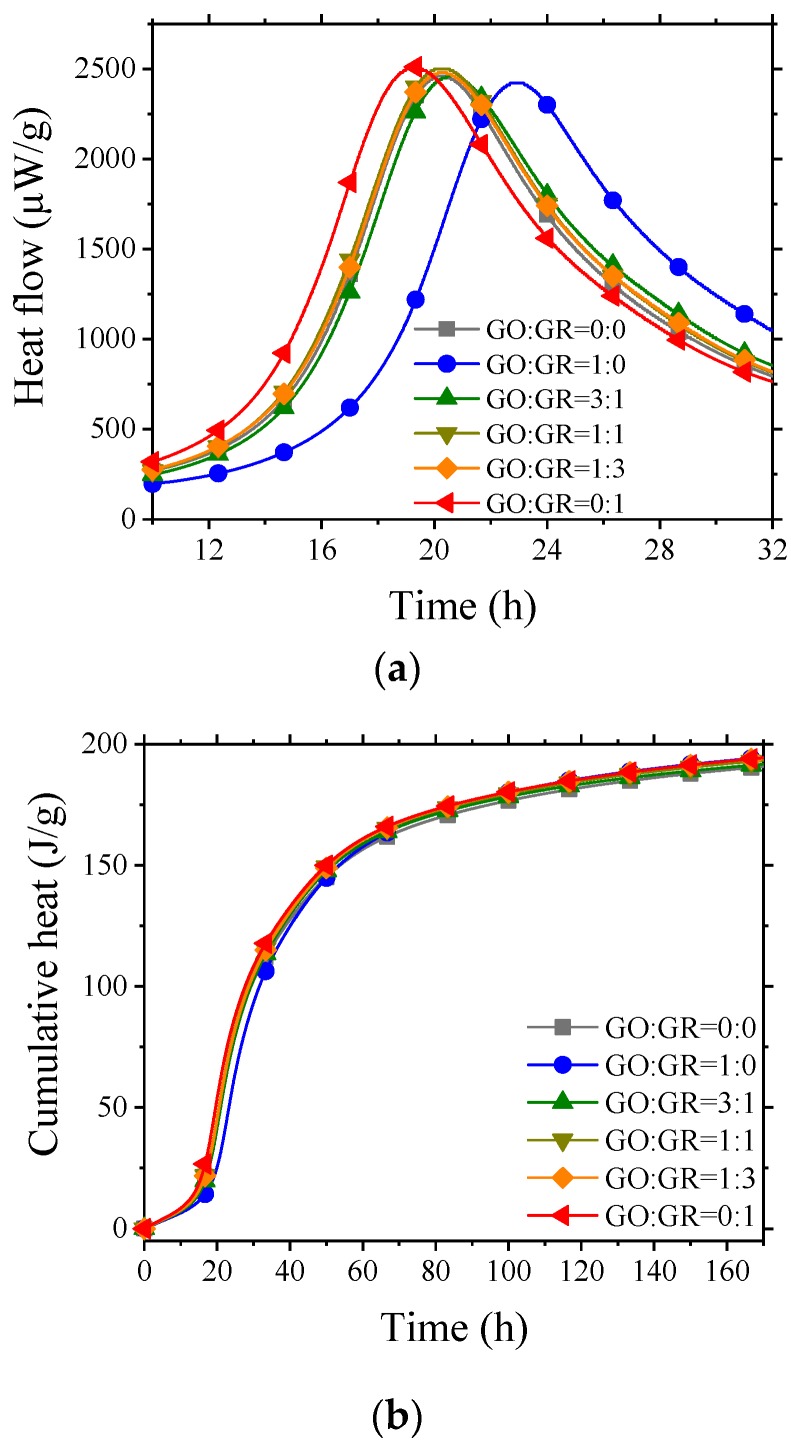
Calorimetry curves of cement with GO/GR hybrid. (**a**) Heat flow; (**b**) Cumulative heat.

In order to investigate the influence of GO/GR hybrid on the early hydration of cement, the heat released during 7 days of hydration was measured. The results are presented in Figure 6. It can be seen that the position of exothermic peak varied with the ratio of GO to GR. The exothermic peak of the specimen prepared with only GR appeared first, and while exothermic peak of the specimen prepared with only GO was delayed by approximately 5 h. The peaks of the other specimens are located in between these two peaks. This indicates that GO and GR had different effects on the early hydration of mortar. GR tended to enhance the early hydration while GO suppresses the early hydration process. Therefore, the specimen prepared with only GR performed slightly better in terms of 3-day compressive strength when compared with specimen prepared with only GO as shown in Figure 4b.

It was also found that, the cumulative heat released by all the specimens showed no obvious difference up to the age of 7 days. However, the 3-day and 7-day compressive strength of specimens with GO/GR hybrid significantly increased (Figure 4b) compared with that of control specimen and specimen prepared with only GO or GR. This indicates that the strength enhancement by hybrid might not only depend on the degree of cement hydration.

### 3.5. Porosity

**Figure 7:**
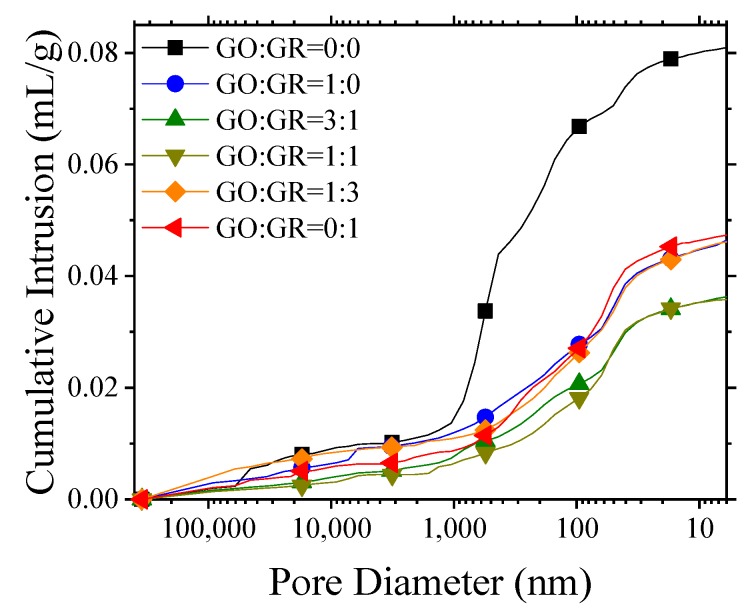
Pore size distribution of mortars with varied GO/GR hybrid at the age of 28 days.

The porosity of mortars prepared with GO/GR hybrid was measured by using MIP at the age of 28 days. The results are presented in Figure 7 and Table 5. In comparison to control specimens, the pore structure of mortars prepared with GO/GR hybrid was significantly improved with total porosity reduced by up to 50.83%, average pore diameter decreased by up to 50.33% and gel-pores smaller than 50 nm increased by 155.79%. Therefore, the compressive strength in Figure 4b was observed to have been significantly enhanced compared to that of control specimen.

In comparison to specimen prepared with only GO and GR, the reduction in porosity and the slight increase in the fraction of gel-pores smaller than 50 nm in GO/GR hybrid cement mortar explains the enhancement of compressive strength of the mortars prepared with GO/GR hybrid as presented earlier in Figure 4b. It is pertinent to mention here that the fraction of gel-pores smaller than 50 nm increased slightly, indicating the slight reduction in the pores larger than 50 nm. Moreover, it was noticed that the relationship between the pore structure and flexural strength is not significant.

## 4. Discussion

In this study, the mechanism and mechanical properties of mortars incorporating GO/GR hybrid were investigated. The flexural strength of cement mortar is mainly determined by the dosage and dispersion of GR since in comparison to GR, the mechanical properties of GO could be speculated to decrease due to the disturbance by sp^3^ carbons [35,36]. As a result, the flexural strength of mortars prepared with only GR performed better than the specimen prepared with only GO especially at the age of 28 days (Figure 4a) when a relatively strong connection was built between GR and cement paste.

When the GO and GR were mixed and added into mortar, the flexural strength of mortar was further improved. It is believed to be due to the dispersibility of GR, that is, the incorporation of GO promoted the dispersion of GR and in turn, reduced the defects caused by the aggregation of GR in mortar. Thus further improvement in the mechanical properties was observed.

In terms of compressive strength, the degree of hydration would influence the strength development, showing higher strength of GR reinforced mortar when compared to GO reinforced mortar (Figure 4b). However, for the mortars incorporated GO/GR hybrid, further enhancement of compressive strength is more related to the dispersion of GO/GR hybrid in water. Fully dispersed GO and GR filled the pores of mortar [37], and thus reduced the total porosity and improved the pore structure of mortar (increase of fraction of pores larger than 50 nm).

Therefore, combining GO with GR and adding them to mortar would benefit further in terms of enhancing the mechanical properties and would contribute to reduce the cracking of mortar. The utilization of GO/GR hybrid would also reduce the cost when compared with the utilization of only GR in cement mortar. Although the mechanical properties of GO are not as good as GR, still GO can be used as a dispersant to improve the dispersion of GR. It is believed that by combining with other nano-materials, GO would improve the overall performance of the related hybrid during application.

## 5. Conclusions

In this research, GO and GR hybrid was used as an additive in cement mortar to improve the dispersion of GR in mortar and to improve the mechanical properties of mortar. Following are the conclusions of this research.

1. GR was significantly dispersed in water when GO was incorporated. When the total amount of GO and GR was constant, the increase of GO dosage was beneficial for the dispersion of GR, which in turn, was beneficial for the mechanical properties of the cement mortar. Meanwhile, the decreased GR content in the hybrid with the increase of GO content was considered to reduce the mechanical properties of mortar. The tradeoff between the two factors resulted in the best performance of mortar at certain GO to GR ratios, such as 1:1 or 3:1.
2. The enhancement of flexural strength is related to the amount of GR addition and its dispersion in GO. Therefore, it is believed that the mechanical properties of mortar could be further increased if a more effective dispersant for GR can be found and used.
3. In addition to dispersibility, the enhancement in compressive strength is more closely related to the porosity and pore size distribution of cement mortar after hydration, while the enhancement in compressive strength is less related to the degree of hydration for mortars incorporating GO/GR hybrid.

## Figures and Tables

**Table 1:** The mass ratios of GO and grapheme (GR) in GO/GR hybrid and corresponding concentrations in mortar (%, relative to the mass of cement).

GO:GR	GO (%)	GR (%)
0:0	0	0
1:0	0.08	0
3:1	0.06	0.02
1:1	0.04	0.04
1:3	0.02	0.06
0:1	0	0.08

**Table 2:** Properties of cement.

Finesse (%)	Density (g/cm^3^)	Specific Surface Area (m^2^/kg)	Consistency (%)
0.08	3.15	346	24.8

**Table 3:** Flexural strength of cement mortars incorporating GO/GR hybrid.

GO:GR	Flexural Strength (MPa)			Increment (%)		
	3 d	7 d	28 d	3 d	7 d	28 d
0:0	6.12	8.48	9.60	–	–	–
1:0	6.80	8.72	10.20	11.11	2.83	6.25
3:1	7.52	9.62	11.29	22.88	13.44	17.60
1:1	7.66	10.06	12.55	25.16	18.63	30.73
1:3	7.46	9.88	11.82	21.90	16.51	23.13
0:1	6.77	8.70	11.73	10.62	2.59	22.19

**Table 4:** Compressive strength of cement mortars incorporating GO/GR hybrid.

GO:GR	Compressive Strength (MPa)			Increment (%)		
	3 d	7 d	28 d	3 d	7 d	28 d
0:0	32	43.1	56.4	–	–	–
1:0	36.5	47.6	63.3	14.06	10.44	12.23
3:1	44.2	56.2	66.4	38.13	30.39	17.73
1:1	43.4	53.4	67.4	35.63	23.90	19.50
1:3	40.7	55.2	65.2	27.19	28.07	15.60
0:1	39.2	49.0	64.8	22.50	13.69	14.89

**Table 5:** Pore size distribution of mortars with GO/GR hybrid.

GO:GR	Porosity (%)	Average Pore Diameter (nm)	Proportion of Pore Diameter under 50 nm (%)
0:0	16.78	119.15	15.54
1:0	10.35	59.18	36.83
3:1	8.31	61.8	38.86
1:1	8.25	64.52	39.75
1:3	10.33	60.19	38.90
0:1	10.56	70.38	32.51
